# Study of the Effect of Memantine on Negative Sign in Patients with Schizophrenia and Schizoaffective Disorders

**Published:** 2018

**Authors:** Maria Tavakoli-Ardakani, Hamide Abbaspour, Abdollah Farhadi Nasab, Azadeh Mazaheri Meibodi, Ali Kheradmand

**Affiliations:** a *Clinical pharmacy, Shahid Beheshti University of Medical Sciences, Tehran, Iran. *; b Students′ Research Committee, School of Pharmacy, *Shahid Beheshti University of Medical Sciences, Tehran, Iran.*; c *Psychiatry, Behavioral Sciences Research Center, Shahid Beheshti University of Medical Sciences, Tehran, Iran.*; d *Psychiatry, Shahid Beheshti University of Medical Sciences, Tehran, Iran.*

**Keywords:** Schizophrenia, memantine, biomarkers, Interleukin-6, Tumor Necrosis Factor-alpha, C - Reactive Protein

## Abstract

Memantine, an uncompetitive antagonist of glutamate receptor of the N-methyl-D-aspartate type is approved for the treatment of moderate to severe Alzheimer disease (1). A growing body of evidence supports a link between the glutamatergic neurotransmission and schizophrenia (2). The aim of this study was to examine the efficacy and safety of memantine as an adjunctive treatment for antipsychotics in patient with psychopathology of schizophrenia and schizoaffective. In this study, we assessed the effect of memantine on the pro-inflammatory cytokines such as IL6, TNFα and CRP. In this double-blind, placebo-controlled study, participants were assigned to receive (5-20 mg/day) memantine (n = 29) or placebo (n = 29), in addition to continuing treatment with antipsychotic for 12 weeks. The primary efficacy measure was the level of pro-inflammatory cytokines (TNFA, IL6, CRP). Safety was assessed by means of physical examination, clinical laboratory evaluation, recording of adverse event (AEs), and measure of extrapyramidal symptoms. At end point, comparison of biomarkers (IL6, TNFα and CRP) in two groups before and after treatment showed a significant decrease of TNFα (P < 0.001), but the difference was not significant in CRP and IL6 level (p = 0.92 and p = 0.77, respectively). The frequency of serious AEs in the memantine vs. placebo group was similar.

## Introduction

Schizophrenia is a serious mental illness. It accounts for 1.1% of the total disability-adjusted life years worldwide and 2.8% of the years lived with disability worldwide. It affects about 1% of the world population (3), with similar rates across different countries, culture and sexes. It is one of the most severe mental injuries that begins at the early-old age and affect men and women with the same ratio. The age of onset tends to be between 16 to 30 years and the illness can be present throughout the person’s adult life. The cause is unknown but is believe that genetic factors, early environmental and social factors can contribute. Total therapeutic and indirect costs of the patients reach about 50 billion dollars a year. On the other hand, schizophrenia is a chronic disease and affected patients occupy about 50% of the beds of the mental hospitals (4). 

A person diagnosed with schizophrenia often presents with a combination of positive (i.e. hallucination, delusions, catatonic behavior), negative (i.e. apathy, low motivation, social withdrawal) and cognitive (i.e. memory deficits, difficulty in integrating thought, behavior and feelings) symptom which lead to problems in social and occupational functioning and self – care (5, 6). Antipsychotic drugs are the major treatments for schizophrenia and are divided into two main groups :1) dopamine receptor antagonist and 2) antagonist associated with serotonin and dopamine, but these medication only eliminate the symptom of the disorder and do not cure the disease itself. Despite proven efficacy, their use is associated with a high prevalence of residual morbidity, such as cognitive deficits and the persistence of positive and negative symptoms (7). Consequently, clinicians often treat patients with chronic schizophrenia with multiple medications, including combinations of antipsychotics and anticonvulsants. Therefore, there is an urgent need for development of more effective treatment for schizophrenia for use as mono therapies or as adjuncts to antipsychotic drugs.

There are various hypotheses regarding the pathogenesis of this disease. For decades, the mainstay neurochemical hypothesis behind schizophrenia has been dopamine dysfunction. This suggests that excess dopaminergic neurotransmission, particularly in the striatal brain region and dopaminergic deficits in the pre-frontal brain region may be responsible for causing the positive and negative symptom of schizophrenia (8). One of the hypothesis about the pathogenesis of schizophrenia is the immune system interference (9, 10). The inflammatory hypothesis of schizophrenia is not new, but recently, it has regained interest because more data suggest a role of the immune system in the pathogenesis of schizophrenia. If increased inflammation of the brain contributes to the symptoms of schizophrenia, reduction of the inflammatory status could improve the clinical picture. Lately, several trials have been conducted investigating the potential of anti-inflammatory agents to improve symptoms of schizophrenia. Emerging data indicate a direct involvement of the glutamatergic system in the pathophysiology of schizophrenia core system (11). Complementary to the dopamine hypothesis, the hypofunction of the inotropic glutamate NMDA has been proposed as a model of schizophrenia in humans. The glutamatergic hypothesis of schizophrenia postulates that the dysfunction of neurotransmission mediated by the NMDA might represent a primary deficit in this disorder. The ability of phencyclidine, ketamine, and other NMDA antagonists to induce schizophrenia – like symptoms in healthy volunteers and to exacerbate psychosis in schizophrenic patients is the most compelling evidence of a relationship between NMDA function and schizophrenia (12). 

**Table 1:** demographic and clinical characteristic of the study participants at baseline.

characteristics	Placebo group(n=29 )	Memantine group (n=29)	*P *value
Age (years )	39 ± 12.58	37±10.91	0.59
Gender ( female % )	37 %	51 %	0.43
Smoking – yes (%)	41 %	44 %	0.79
BMI ( kg/m2)	26.96 ±2.86	27.47 ±2.79	0.35
Marital status ( married %)	10 %	20 %	0.47
Concurrent disease- have (%)	13 %	17 %	1.00
Use of anticholinergic drugs ( % subjects )	93.5 %	90.9 %	1.00
Antipsychotic agent Typical (%)	43 %	44 %	0.78
Disease type (% subjects ) Schizophrenia Schizoaffective	55%	58 %	0.79
Daily chlorpromazine equivalent dose ( mg )	44 %	41.3%	0.79
Clinical rating scale scores PANSS subscale	1261.7±1078.6	986.4±831.6	0.57
Negative	33.72±7.57	34.62±4.01	0.91
Positive	34.65±4.56	33.65±4.06	0.38
General	75.44±8.1	76.27±7.4	0.69
Total	139.13±12	137.44±10.9	0.83

**Table 2:** effect of memantine compared with placebo on psychiatric assessment score over the 12-week study period.

	Memantine ( n = 29 ) Mean ± SD		placebo (n = 29 ) Mean ± SD		*p *value
	Week 4	Week 12	Week 4	Week 12	
PANSS					
Total	130 ± 14.11	34.72 ± 20.07	138 ± 13.02	6.76 ± 14.86	≤ 0.001
Positive subscale	28.41 ± 4.56	7.17 ± 6.32	31 ± 5.25	4.17 ± 6.25	0.07
Negative subscale General psychopathology	32.34 ± 4.49	10.48 ± 5.54	33.65 ± 4.28	0.45 ± 5.63	≤ 0.001
subscale	70 ± 8.7	17.07 ± 11.30	73.37 ± 8.08	2.13 ± 8.5	≤ 0.001

**Table 3:** effect of memantine compared with placebo on biomarkers level over the 12-week study period.

	Memantine ( n = 29 ) Mean ± SD		placebo (n = 29 ) Mean ± SD		*p *value
	Week 4	Week 12	Week 4	Week 12	
IL6	3.52 ± 4.2	0.85 ± 4.83	4.72 ± 8.4	0.61 ± 3.34	0.37
TNF-α	4.87 ± 4.78	2.13 ± 4.59	2.80 ± 0.97	-0.92 ± 1.83	≤ 0.001
CRP	12.46 ± 22.39	3.07 ± 16.63	10.61 ± 11.7	2.03 ± 12.14	0.92

**Table 4.** adverse effect of memantine and placebo

	Memantine( n = 29)	placebo (n = 29 )
Dizziness	3 (10 %)	2 (6.8 %)
Diarrhea	2 (6.8 %)	2 (6.8 %)
Constipation	2 (6.8 %)	1 (3.4 %)
Headache	0	1 (3.4 %)
Agitation	9 (31 %)	8 (27 %)
Insomnia	4 (13 %)	5 (17 %)
Total	20 (68 %)	19 (65 %)

Memantine, an uncompetitive N-methyl-D-aspartate (NMDA) receptor antagonist, is approved for the treatment of moderate to severe Alzheimer disease (AD) in many countries worldwide (13, 14). It has been shown to delay cognitive decline in patients with dementia, an effect hypothesized to arise from its ability to block the excessive influx of calcium ions through the channel of the activated NMDA receptor (15).

The rationale for using memantine as an adjunctive therapy for patients with schizophrenia comes from the fact that the glutamatergic system, and specifically hypofunctioning of NMDA receptors, has been implicated in the pathophysiology of schizophrenia, and is thought to mediate several psychopathological components of the disease including psychotic, negative, and cognitive symptoms (16). Schizophrenic symptoms may also be improved by NMDA receptor antagonist, as glutamatergic neurons in the prefrontal cortex are inhibited by NMDA-mediated GABA interneurons. NMDA receptor antagonists have been shown to release this inhibition, producing profound indirect excitation of cortical pyramidal neurons and increased glutamatergic outflow in the cortex. There is more evidence to suggest dysregulation of NMDARs as an important factor in the pathophysiology of schizophrenia. There seem to be an association between NMDA receptors and dopamine (17).

Although NMDA receptors are located throughout the brain, they may play a role in dopamine release via NMDA receptors. This possibly explains that dopaminergic deficits in schizophrenia could also be a result of underlying glutamatergic dysfunction (18). Memantine due to its uncompetitive antagonist and rapid on-and-off kinetics, and neuroprotective properties, may be of use in restoring this dysfunction and providing the required neuroprotection in schizophrenia patients. We anticipate that because of its novel action, it would prove useful in patients with schizophrenia, notably by improving the negative symptoms (19, 20).

Finally, the efficacy of memantine in delaying cognitive decline of patient with AD suggests a potential for treating cognitive impairment, as well as for preventing progression of the illness in schizophrenia (21). 

The aim of this study was to examine the efficacy and safety of memantine as an addition to ongoing therapy with antipsychotic agent in patient with schizophrenia and schizoaffective disorder. 

## Materials and methods

*Trial design*

The study was a 12 week prospective, double blind and placebo controlled trial. Two parallel groups of patients were randomly selected from Tehran Taleghani hospital and these population participated in the study from September 2014 to March 2016.

*Participant and Setting*

Patients were recruited from the Taleghani Hospital of Shahid Beheshti University of Medical Sciences. Informed consent was obtained from all participants before their enrollment. Participants who were eligible for the study met Diagnostic and Statistical Manual of Mental Disorders (DSM-5) criteria for schizophrenia (22). Two groups were well balance in terms of age, sex, years of education and positive and negative symptom scale (PANSS) measures of negative, positive, general and total score (23).

Inclusion criteria was patients aged 18-65 years old, patients will be recruited both from inpatient and outpatient, patient that met DSM-5 criteria for diagnosis of schizophrenia and schizoaffective disorder, competent and willing to give informed consent, stable on medication four weeks prior to baseline, no planned medication change, able to take oral medication and likely to complete the required evaluations, female participants of child bearing age must be willing to use adequate contraceptives for the duration of study and be willing to have a pregnancy test pre-treatment.

Patients that use clozapine and also subjects who met the criteria for a DSM-5 diagnosis of alcohol or substance abuse within the previous month were excluded. Exclusion also include pregnant or lactating women, any change of psychotic medications within the previous four weeks, and patients that has organic brain disease or an active seizure.

**Figure 1:**
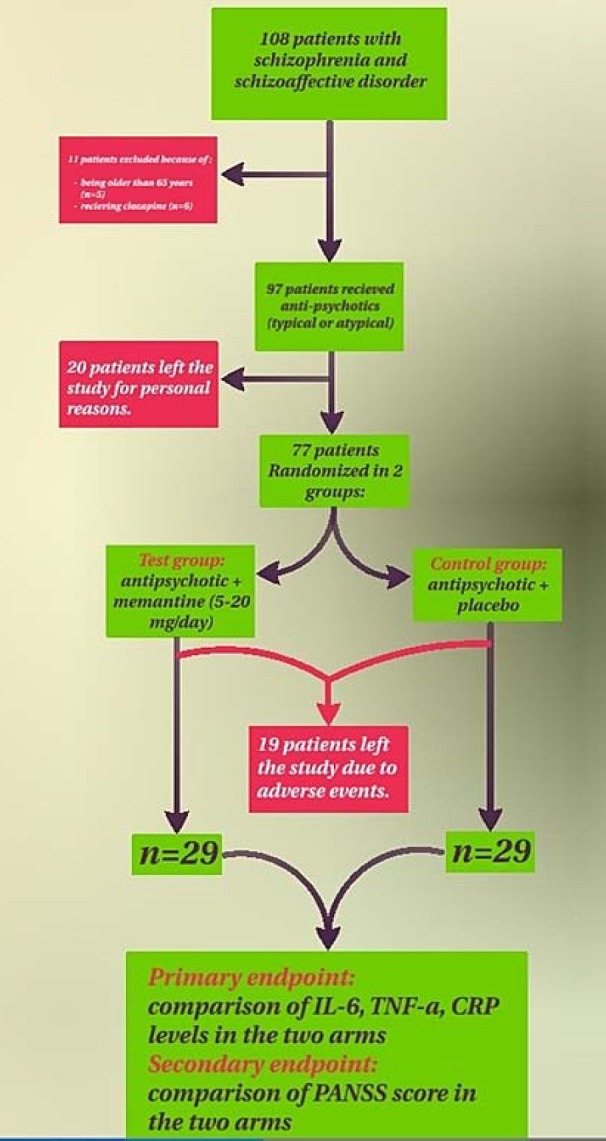
Consort Flowchart.

*Randomization*

Eligible patients were randomized using a randomization table for memantine (5-20 mg/day) or a matching placebo (Sobhan, Rasht, Iran). Both participants and investigators were blind to treatment randomization throughout the study. Participants were instructed not to change their diet and medications during the 12-week study period. The patients can either be on first or second generation antipsychotic medications, as deemed suitable by the responsible psychiatrist, the distribution was approximately 50:50 between the two generations of antipsychotics. Subjects were randomly selected to receive memantine treatment or a placebo, in addition to a fixed daily dose of conventional antipsychotic medication. Subjects in the memantine group received an initial dose of 5 mg/day, which was increased weekly in 5 mg increments until reaching a maximum dose of 20 mg/day. This maximum dose was chosen according to the effective dose established for patients with dementia. The primary (PANSS Score) and secondary (inflammatory biomarkers level such IL6, TNFα and CRP) outcomes were assessed at baseline, and at Weeks 4 and 12. PANSS Score was used to assess the severity of psychiatric symptoms. Venous blood was collected in EDTA-K2 and anticoagulant free tubes for measurement of IL6, TNFα and CRP. Serum was obtained by centrifugation at 3000 x g for 5 min and then kept frozen at -80 °C until the assay i.e., 10 months later. Measurement of IL6, TNFα and CRP was obtained by Enzyme immunometric assay using commercial kit (bioscience biochem, USA) and a sunrise ELISA reader. The checklist for adverse events was completed at Weeks 4 and 12.

*Statistical analysis*

Baseline comparisons between the two groups were performed using the Mann –Whitney U test for continuous variables and Fishers exact test for categorical variables. The efficacy of memantine and placebo were analyzed with repeated measures analysis of variance (ANOVA) using group (memantine vs. placebo) as the between subjects factor and time (baseline, Weeks 4 and 12) as the within-subjects factor. The level of statistical significance was set at *p* ≤ 0.05. 

## Results

*Demographic data*

One hundred and eight patients were screened for inclusion in the study of whom 97 patients were eligible and randomized to memantine or placebo group. Thirty nine out of 97 patients withdrew from the study. The remaining 58 subjects were randomly assigned to memantine (n = 29) or placebo (n = 29) treatment group and completed the duration of 12 weeks trial. Flow chart was used throughout the study (Figure 1).

Table1 presents demographic and baseline data for the study population. No significant differences in demographic or clinical variables were observed between the treatment groups at baseline. Subjects also received typical and atypical antipsychotic drugs in ratio of 50:50, and the mean chlorpromazine – equivalent dose did not differ between treatment groups. Ten patients received a combination of two antipsychotic drugs. 

*Effect of memantine therapy on psychiatric symptom*

PANSS score on subscale of positive at baseline and Weeks 4 and 12 not change in response to memantine treatment. In fact, mean PANSS score did not differ between the memantine and placebo group in this subscale, but negative subscale and general psychopathology was significantly different between the memantine and placebo group (Table 2). 

*Effect of memantine on biomarkers (IL6, TNFα and CRP)*

TNF-α was significantly decrease in memantine group compared to control group after 4 weeks (*p* < 0.001) and at the end of the study (12 weeks) (*p* < 0.001). IL6 and CRP were no different in control compared to memantine group at 4 weeks (*p* = 0.37 and *p* = 0.45, respectively) and after the end of the study (*p* = 0.77 and *p* = 0.92, respectively) (Table 3).

*Adverse event as a result of adjunctive memantine therapy*

Memantine was well tolerated and the incidence of adverse events was similar between groups. In total, 68% of the memantine group and 65% of placebo group experienced adverse events. The most common adverse events were agitation and insomnia, but these symptoms were transient and mild to moderate in intensity. No subject demonstrated serious adverse effects during the study (Table 4). 

## Discussion

The present study investigated the effect of adjunctive memantine therapy in combination with conventional antipsychotic medication in patients with schizophrenia and schizoaffective disorder. Statistically significant differences were observed in the memantine group compared with the placebo group in subscale of negative symptom in PANSS score. Also, TNFα as a pro-inflammatory biomarker significantly decrease with memantine treatment.

Therefore, these results support the hypothesis that adjunctive memantine therapy improves negative symptom in patients with schizophrenia and schizoaffective disorder. 

In one study, Krivey *et al*. reported that memantine did not improve cognitive functioning during a 6-week open –label study. But they reported a 21% decrease in the PANS negative subscale score (24). Liberman *et al*. also reported that memantine adjunctive therapy failed to affect cognitive symptoms (25). Silver *et al*. showed that amantadine, an antiviral agent with indirect dopaminergic agonist and NMDA antagonist action, was associated with improved visuomotor coordination compared to a placebo treatment (26). However, memantine did not show beneficial effects on cognitive functioning. Gama *et al*. reported that adjunctive memantine therapy improved scores on the Brief Psychiatric Rating Scale (BPRS) among patient with schizophrenia (27).

Despite the absence of data regarding the effects of memantine on patients with schizophrenia, memantine, unlike other NMDA receptor antagonists, did not induce psychosis. These observations indicate that memantine may represent a safer alternative for patients with schizophrenia.

Although a recent study reported that memantine adjunctive therapy was associated with adverse treatment effects, fewer adverse events were reported with memantine therapy in patients with schizophrenia in the present study compared with patients with Alzheimerꞌs dementia (AD). Reisberg *et al*. reported adverse events in 84% of memantine-treated AD patients and 87% of placebo-group patients. Agitation and urinary tract infections were the most common adverse events (28).

Tariot *et al.* reported that 78% of AD patients receiving memantine and 72% of those receiving placebo experienced adverse events (29). At least, 5% of the memantine group, double the proportion found in the placebo group, experienced confusion and headaches. However, in the current study, no differences in adverse events were observed between the memantine and placebo groups. This discrepancy between schizophrenia and AD in the occurrence of adverse events might be derived from differences in the physiological characteristics or ages of subjects.

This study has several limitations. First, most subjects were undergoing treatment with anticholinergic drugs during the study period. A large body of evidence from human and non-human animal studies has established that the cholinergic neurotransmitter system is important for attention, memory, and learning. Anticholinergic drugs impair cognitive and information processing both in normal populations and in individuals with schizophrenia (30). Moreover, several lines of evidence indicate that NMDA antagonists interact with cholinergic systems (31), although the interaction between cholinergic and glutamatergic systems is complex and poorly understood.

Secondly, we can hypothesize that the 12-week duration of this study might be too short to warrant any conclusions. Finally, the small sample size may have masked significant effects. Although this study can be regarded as pilot study for a large-scale trial, the implications of the results might be restricted, and further study with a larger sample is needed.

Our findings indicate that adjunctive memantine therapy have beneficial effects on negative symptoms or psychopathology among patients with schizophrenia and schizoaffective disorder. However, future studies that address the limitations described above are required to examine more deeply the effects of memantine on negative symptoms in schizophrenia and schizoaffective disorder.

## References

[1] Millan MJ (2005). N-Methyl-D-aspartate receptors as a target for improved antipsychotic agents: novel insights and clinical perspectives. Psychopharmacology (Berl).

[2] Tsapakis EM, Travis MJ (2002). Glutamate and psychiatric disorders. Adv Psychiatr Treat.

[3] Kirkpatrick B, Buchanan RW, Ross DE, Carpenter W (2001). A separate disease within the syndrome of schizophrenia. Arch. Gen. Psychiatry.

[4] Mueser KT, McGurk SR (2004). Schizophrenia. Lancet.

[5] Anderson NC (2000). Schizophrenia: the fundamental questions. Brain Res. Rev.

[6] Andreasen NC (1982). Negative symptoms in schizophrenia Definition and reliability. Arch. Gen. Psychiatry.

[7] Kirkpatrick B, Buchanan RW, Ross DE, Carpenter W (2001). A separate disease within the syndrome of schizophrenia. Arch. Gen. Psychiatry.

[8] Ralf Brisch, Arthur saniotis, Rainer wolf (2014). The Role of Dopamine in Schizophrenia from a Neurobiological and Evolutionary Perspective. front psychiatry , May.

[9] Körschenhausen D, Hampel H, Ackenheil M, Penning R, Müller Fibrin N (1996). degradation products in postmortem brain tissue of schizophrenics: a possible marker for underlying inflammatory processes. Schizophr. Res.

[10] Müller N, Ackenheil M (1998). Psychoneuroimmunology and the cytokine action in the CNS: implications for psychiatric disorders. Prog. Neuro-Psychopharmacol. Biol. Psychiatry.

[11] Millan MJ (2005). N-Methyl-D-aspartate receptors as a target for improved antipsychotic agents: novel insights and clinical perspectives. Psychopharmacology (Berl).

[12] Olney JW, Labruyere J, Wang G, Wozniak DF, Price MT, Sesma MA (1991). NMDA antagonist neurotoxicity: mechanism and prevention. Science.

[13] Tariot PN, Farlow MR, Grossberg GT, Graham SM, McDonald S, Gergel I (2004). Memantine treatment in patients with moderate to severe Alzheimer disease already receiving donepezil: a randomized controlled trial. JAMA.

[14] Reisberg B, Doody R, Stoffler A, Schmitt F, Ferris S, Mobius HJ (2003). Memantine in moderate-to-severe Alzheimerꞌs disease. N Engl J Med.

[15] Schwartz TL, Sachdeva S, Stahl SM (2012). Genetic data supporting the NMDA glutamate receptor hypothesis for schizophrenia. Current pharmaceutical design.

[16] Lieberman JA, Papadakis K, Csernansky J, Litman R, Volavka J, Jia XD (2009). A randomized, placebo-controlled study of memantine as adjunctive treatment in patients with schizophrenia. Neuropsychopharmacology.

[17] Gama CS, Antunes P, Moser C, Belmonte-de-Abreu PS (2005). Memantine as an adjunctive therapy for schizophrenia negative symptoms. Rev Bras Psiquiatr.

[18] Javitt DC (2010). Glutamatergic theories of schizophrenia. The Israel Journal of Psychiatry and Related Sciences.

[19] Maekawa M, Namba T, Suzuki E, Yuasa S, Kohsaka S, Uchino S (2009). NMDA receptor antagonist memantine promotes cell proliferation and production of mature granule neurons in the adult hippocampus. Neuroscience Research.

[20] Schwartz TL, Sachdeva S, Stahl SM (2012). Genetic data supporting the NMDA glutamate receptor hypothesis for schizophrenia. Current pharmaceutical design.

[21] Rogawski MA, Wenk GL (2003). The neuropharmacological basis for the use of memantine in the treatment of Alzheimerꞌs disease. CNS Drug Rev.

[22] First MB, Spitzer RL, Gibbon M, Williams JB (1997). Structured Clinical Interview for DSM-V Axis I Disorders (SCID-I).

[23] Kay SR, Opler LA, Lindenmayer JP (1988). Reliability and validity of the positive and negative syndrome scale for schizophrenics. Psychiatry Res.

[24] Krivoy A, Weizman A, Laor L, Hellinger N, Zemishlany Z, Fischel T (2008). Addition of memantine to antipsychotic treatment in schizophrenia in patients with residual symptoms: a preliminary study. Eur Neuropsychopharmacol.

[25] Lieberman JA, Papadakis K, Csernansky J, Litman R, Volavka J, Jia XD (2009). A randomized, placebo-controlled study of memantine as adjunctive treatment in patients with schizophrenia. Neuropsychopharmacology.

[26] Silver H, Goodman C, Isakov V, Knoll G, Modai I (2005). A double-blind, cross-over comparison of the effects of amantadine or placebo on visuomotor and cognitive function in medicated schizophrenia patients. Int Clin Psychopharmacol.

[27] Gama CS, Antunes P, Moser C, Belmonte-de-Abreu PS (2005). Memantine as an adjunctive therapy for schizophrenia negative symptoms. Rev Bras Psiquiatr.

[28] Reisberg B, Doody R, Stoffler A, Schmitt F, Ferris S, Mobius HJ (2003). Memantine in moderate-to-severe Alzheimer’s disease. N Engl J Med.

[29] Tariot PN, Farlow MR, Grossberg GT, Graham SM, McDonald S, Gergel I (2004). Memantine treatment in patients with moderate to severe Alzheimer disease already receiving donepezil: a randomized controlled trial. JAMA.

[30] Friedman JI (2004). Cholinergic targets for cognitive enhancement in schizophrenia: focus on cholinesterase inhibitors and muscarinic agonists. Psychopharmacology (Berl).

[31] Macro LA, Joshi RS, Brown C, Aldes LD, Chronister RB (1988). Effects of cholinergic and anticholinergic drugs on ketamine-induced linguopharyngeal motor activity. Psychopharmacology (Berl).

